# Multistage closure of a congenital extrahepatic portosystemic shunt

**DOI:** 10.1186/s42155-021-00267-x

**Published:** 2021-11-18

**Authors:** João Facas, Manuel Cruz, João Filipe Costa, Alfredo Agostinho, Paulo Donato

**Affiliations:** 1 Medical Imaging Department, Coimbra University Hospital, Praceta Prof. Mota Pinto, Coimbra, Portugal; 2 Faculty of Medicine of the University of Coimbra, Azinhaga de Santa Comba, Coimbra, Portugal

**Keywords:** Congenital extrahepatic portosystemic shunt, Abernethy, Atrial septal defect occluder, Transjugular intrahepatic portosystemic shunt, Balloon occlusion venography

## Abstract

**Background:**

Congenital extrahepatic portosystemic shunts (CEPS) are rare shunts connecting the extrahepatic portal system with the inferior vena cava. Shunt dimensions and the risk of portal hypertension determines the closure strategy. Endovascular treatment is indicated for single stage occlusion of longer length shunts, whereas the remaining shunt types are preferentially surgically occluded. Herein we describe the technical details of a novel endovascular treatment for short length CEPS.

**Case presentation:**

A 15-years-old male with a short length CEPS complicated with multinodular liver disease was submitted to a multistage closure, as indicated by the high portal pressure values during shunt balloon occlusion venography. Initially a transjugular intrahepatic portosystemic shunt (TIPS) was created and the CEPS occluded with an atrial septal defect occluder. In a second procedure the TIPS was embolized with a flow reductor stent and an amplatzer vascular plug II. At a 1 year follow up the liver nodules size reduced, the patient remains asymptomatic, and the shunt adequately closed.

**Conclusion:**

This paper outlines the potential use of a TIPS and an atrial septal defect occluder combination in complex CEPS, supporting its usage as an alternative to the standard surgical treatment.

Level of Evidence: Level 4, Case report.

## Background

Congenital extrahepatic portosystemic shunts (CEPS), also known as Abernethy malformations, are rare shunts connecting the portal system proximal to its bifurcation with the supra-renal inferior vena cava (IVC) (Alonso-Gamarra et al., 2011). CEPS are further divided into type I, an end-to-side shunt without intrahepatic portal system, and type II, a side-to-side shunt with an underdeveloped intrahepatic portal system, where both surgical and endovascular closure is feasible (Franchi-Abella et al., 2018).

The portal blood bypass results in a liver perfusion disarrangement and an impaired first pass effect, responsible for the clinical spectrum in untreated CEPS which includes nodular liver disease, hyperammonemia, encephalopathy and hepatopulmonary syndrome. When complications are present closure is recommended, as CEPS spontaneous closure is uncommon (Baiges et al., 2020; Rajeswaran et al., 2020).

Treatment selection is based on the risk of portal hypertension and shunt dimensions. A venography is performed, and the shunt temporarily occluded with a balloon to determine portal pressure before and after shunt occlusion. If portal pressure after shunt occlusion is above 24 mmHg or portal pressure gradient before/after shunt occlusion is above 9 mmHg a multistage closure must be selected due to the increased risk of portal hypertension. As for shunt dimensions, it’s a subjective classification where a shunt is considered short length when the risk of non-target embolization and turbulent flow with standard coils or plugs precludes a safe procedure. Endovascular treatment is indicated for long length single stage closures, whereas short length shunts and multistage occlusions are preferentially surgically approached with ligation, clips placement, caval partition or banding, often in combination with endovascular treatment. (Baiges et al., 2020; Bruckheimer et al., 2013; Rajeswaran et al., 2020).

We report a multistage occlusion of a short length CEPS occluded with an atrial septal defect plug in combination with a transjugular intrahepatic portosystemic shunt (TIPS) creation, that was later occluded with a flow reductor stent and an amplatzer vascular plug II.

## Case presentation

A 15-year-old male was referred to a gastroenterology consultation due to increased total bilirubin (71,8 μmol/L), alanine transaminase (65 IU/L) and aspartate transaminase (55 IU/L) levels. Ammonia levels were normal. The lab tests were required before isotretinoin initiation for acne disease. The patient was asymptomatic and had no relevant past history.

**Fig. 1:**
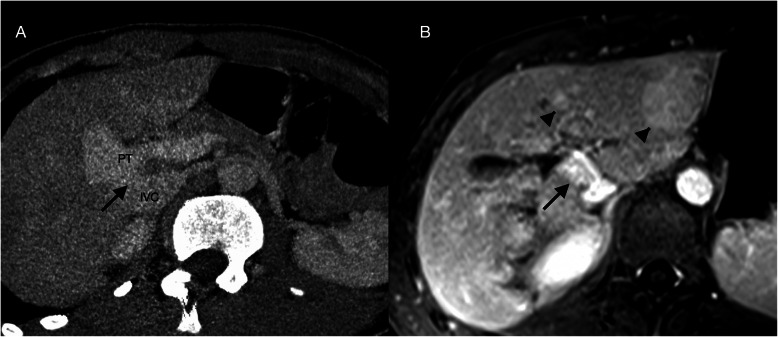
Type II congenital extrahepatic portosystemic shunt. **A**) CT mutiplanar reconstruction. A wide and short length shunt (black arrow) is seen connecting the inferior vena cava (IVC) and the portal trunk (PT). **B**) Axial equilibrium phase T1-weighted MR image with gadobutrol shows two slightly hyperintense nodules. These were also hyperintense on arterial and portal phases, compatible with the diagnosis of regenerative nodules (arrow heads)

An abdominal ultrasound identified multiple hyperechoic nodules and a direct communication between the portal trunk and the IVC with hypoplastic portal branches. Subsequent CT and MRI confirmed the presence of a type II CEPS, measuring 16 mm in diameter and 4 mm in length, and the nodules were characterized as probable regenerative nodules (Fig. 1).

The patient underwent a portocaval venography and shunt temporarily occluded with a balloon. Portal pressure values increased from 16 mmHg to 28 mmHg after occlusion (12 mmHg gradient). Given these values a progressive closure of the CEPS was planned.

**Fig. 2:**
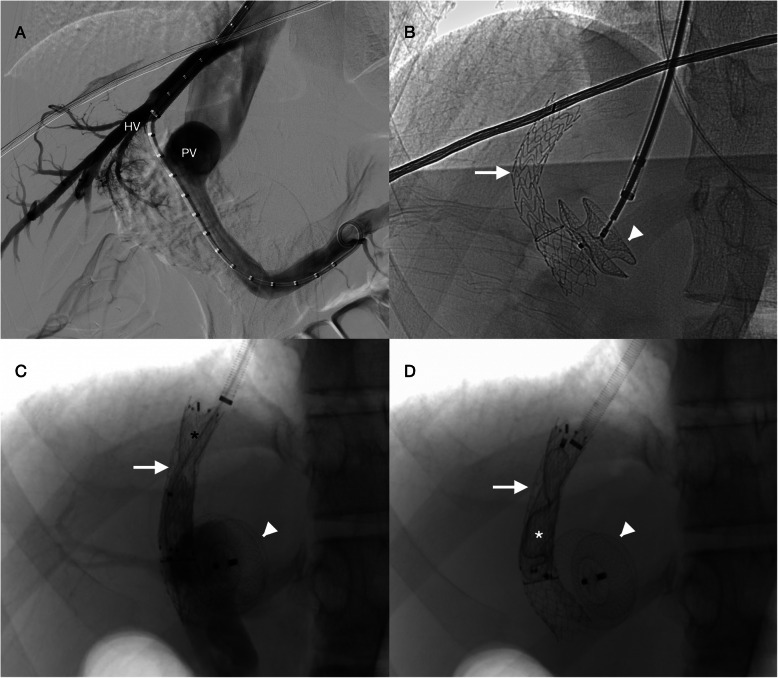
Multistage closure of a type II congenital extrahepatic portosystemic shunt. **A**) and **B**) In the first procedure an atrial septal defect plug (white arrowhead) was deployed in the shunt after a transjugular intrahepatic portosystemic shunt creation (white arrow) between the middle hepatic vein (HV) and the right portal vein (PV). **C**) During the second procedure, a balloon occlusion portal venography was performed after the deployment of a flow restrictor stent (black asterisk). There was an 8 mmHg portal pressure increase to 14 mmHg. **D**) As portal pressure remained low, the TIPS was ultimately closed using a vascular plug (white asterisk)

In the initial procedure, through a transjugular access, a TIPS was created between the middle hepatic vein and right portal vein (GORE® VIATORR® TIPS Endoprosthesis 8-10 × 50 + 20 mm). Using the 10F TIPS sheath the CEPS was occluded with a 16 mm atrial septal defect occluder (Amplatzer™ septal occluder, Abbott®), matching the CEPS diameter previously measured on CT. The TIPS was created before the CEPS occlusion to avoid the risks of an unsuccessful TIPS creation (Fig. 2A and B). TIPS patency and CEPS closure were assessed with Doppler ultrasound.

A second procedure was performed forty days later with downstaging of the TIPS caliber using a flow reductor stent (14/4/14x40mm sinus-reduction stent, Optimed®), resulting in a minor increase in portal pressure from 6 to 8 mmHg (Fig. 2C). Temporary shunt balloon occlusion was repeated with portal pressure increasing to 14 mmHg. According to these portal pressure values a decision was made to definitively occlude the TIPS using a 14 mm Amplatzer™ Vascular Plug II (Fig. 2D). The patient was started on 40 mg/day of enoxaparin.

Likewise, TIPS and CEPS closure were accessed with Doppler ultrasound. At 2 weeks follow up a non-occlusive peripheral thrombus was identified in the left portal branch and enoxaparin dosage upscaled to 60 mg/day. The thrombus resolved 3 months later and enoxaparin was withdrawn. At a 1 year follow up the TIPS and CEPS remained occluded, liver nodules size decreased, the patient is asymptomatic and did not develop additional complications. Total bilirubin (68,4 μmol/L), alanine transaminase (68 IU/L) and aspartate transaminase (39 IU/L) did not decrease to normal levels.

## Discussion

The literature on endovascular multistage closure of type II CEPS is scarce. For longer length shunts custom-made flow restrictors (Bruckheimer et al., 2013; Roggen et al., 2018) and consecutive intra shunt stent deployment and epithelization (Eroglu et al., 2004) have been utilized. However, to our knowledge, only one case of a shorth length multistage closure has been reported, which combined an IVC stent with a temporary TIPS (Chick et al., 2017).

Herein we report a novel technique with the combinations of an atrial septal defect occluder with a temporary TIPS. The off-label usage of atrial septal defect occluders have been previously reported in single stage occlusions of short length CEPS (Alharbi et al., 2017; Kuo et al., 2010). They have a reduced risk of migration, with larger outer disks in the portal vein and vena cava acting as a lock mechanism, and a reduced risk of turbulent flow due to their relatively low thickness. As for the TIPS, it both decreases the shunted portal blood, as a result of its smaller diameter in relation to the CEPS, and acts as a conduit for further embolization. In multistage closure measuring the portal pressure during shunt balloon occlusion is critical not only in the initial evaluation but also between procedures, as it dictates whether a partial or a definitive occlusion is indicated.

Regarding patient follow up the hepatic nodules size decreased and no further complications developed. This is consistent with the literature in which shunt closure is effective in both management and prevention of complications, particularly important considering the risk of developing fatal complications such as pulmonary hypertension and hepatocellular carcinoma (Baiges et al., 2020).

## Conclusion

This report highlights the feasibility of endovascular closure in complex CEPS, supporting its usage as an alternative to the standard surgical treatment. However, since it’s a single case report, further studies are necessary to validate its efficiency and safety.

## Data Availability

Not applicable.

## Abbreviations

CEPS: Congenital extrahepatic portosystemic shunt
IVC: Inferior vena cava
TIPS: Transjugular intrahepatic portosystemic shunt

## Declarations

Ethical approval

For this type of study, formal consent is not required.

## References

[CR1] Alharbi A, Abdulrahman S, AlOtaibi M, Alomrani A, Arabi M (2017). Congenital extrahepatic portosystemic shunt embolization with the use of a duct Occluder in a neonate with liver dysfunction and Hyperammonemia. J Vasc Interv Radiol.

[CR2] Alonso-Gamarra E, Parrón M, Pérez A, Prieto C, Hierro L, López-Santamaría M (2011). Clinical and radiologic manifestations of congenital extrahepatic portosystemic shunts: a comprehensive review. Radiographics.

[CR3] Baiges A, Turon F, Simón-Talero M, Tasayco S, Bueno J, Zekrini K, Plessier A, Franchi-Abella S, Guerin F, Mukund A, Eapen CE, Goel A, Shyamkumar NK, Coenen S, De Gottardi A, Majumdar A, Onali S, Shukla A, Carrilho FJ (2020). Congenital extrahepatic portosystemic shunts (Abernethy malformation): an international observational study. Hepatology.

[CR4] Bruckheimer E, Dagan T, Atar E, Schwartz M, Kachko L, Superina R, Amir G, Shapiro R, Birk E (2013). Staged transcatheter treatment of portal hypoplasia and congenital portosystemic shunts in children. Cardiovasc Intervent Radiol.

[CR5] Chick JFB, Reddy SN, Yu AC, Kelil T, Srinivasa RN, Cooper KJ, Saad WE (2017). Three-dimensional printing facilitates successful endovascular closure of a type II Abernethy malformation using an Amplatzer atrial septal Occluder device. Ann Vasc Surg.

[CR6] Eroglu Y, Donaldson J, Sorensen LG, Vogelzang RL, Melin-Aldana H, Andersen J, Whitington PF (2004). Improved neurocognitive function after radiologic closure of congenital portosystemic shunts. J Pediatr Gastroenterol Nutr.

[CR7] Franchi-Abella S, Gonzales E, Ackermann O, Branchereau S, Pariente D, Guérin F (2018). Congenital portosystemic shunts: diagnosis and treatment. Abdom Radiol.

[CR8] Kuo MD, Miller FJ, Lavine JE, Peterson M, Finch M (2010). Exploiting phenotypic plasticity for the treatment of Hepatopulmonary shunting in Abernethy malformation. J Vasc Interv Radiol.

[CR9] Rajeswaran S, Johnston A, Green J, Riaz A, Thornburg B, Mouli S, Lautz T, Lemoine C, Superina R, Donaldson J (2020). Abernethy malformations: evaluation and management of congenital portosystemic shunts. J Vasc Interv Radiol.

[CR10] Roggen M, Cools B, Maleux G, Gewillig M (2018). A custom-made percutaneous flow-restrictor to manage a symptomatic congenital Porto-systemic shunt in an infant. Catheter Cardiovasc Interv.

